# Autophagy Activation Promoted by Pulses of Light and Phytochemicals Counteracting Oxidative Stress during Age-Related Macular Degeneration

**DOI:** 10.3390/antiox12061183

**Published:** 2023-05-30

**Authors:** Roberto Pinelli, Michela Ferrucci, Francesca Biagioni, Caterina Berti, Violet Vakunseth Bumah, Carla Letizia Busceti, Stefano Puglisi-Allegra, Gloria Lazzeri, Alessandro Frati, Francesco Fornai

**Affiliations:** 1 SERI, Switzerland Eye Research Institute, 6900 Lugano, Switzerland; roberto@seri-lugano.ch (R.P.); backoffice@seri-lugano.ch (C.B.); 2 Human Anatomy, Department of Translational Research and New Technologies in Medicine and Surgery, University of Pisa, 56126 Pisa, Italy; michela.ferrucci@unipi.it (M.F.); gloria.lazzeri@unipi.it (G.L.); 3 IRCCS, Istituto di Ricovero e Cura a Carattere Scientifico, Neuromed, 86077 Pozzili, Italy; francesca.biagioni@neuromed.it (F.B.); carla.busceti@neuromed.it (C.L.B.); stefano.puglisiallegra@neuromed.it (S.P.-A.); alessandro.frati@neuromed.it (A.F.); 4 Department of Chemistry and Biochemistry, College of Sciences, San Diego State University, San Diego, CA 92182, USA; vbumah@sdsu.edu; 5 Department of Chemistry and Physics, University of Tennessee, Martin, TN 38237, USA

**Keywords:** autophagolysosomes, drusen, visual acuity, curcumin, lutein, resveratrol, bilberry, amber light, red light, blue light

## Abstract

The seminal role of autophagy during age-related macular degeneration (AMD) lies in the clearance of a number of reactive oxidative species that generate dysfunctional mitochondria. In fact, reactive oxygen species (ROS) in the retina generate misfolded proteins, alter lipids and sugars composition, disrupt DNA integrity, damage cell organelles and produce retinal inclusions while causing AMD. This explains why autophagy in the retinal pigment epithelium (RPE), mostly at the macular level, is essential in AMD and even in baseline conditions to provide a powerful and fast replacement of oxidized molecules and ROS-damaged mitochondria. When autophagy is impaired within RPE, the deleterious effects of ROS, which are produced in excess also during baseline conditions, are no longer counteracted, and retinal degeneration may occur. Within RPE, autophagy can be induced by various stimuli, such as light and naturally occurring phytochemicals. Light and phytochemicals, in turn, may synergize to enhance autophagy. This may explain the beneficial effects of light pulses combined with phytochemicals both in improving retinal structure and visual acuity. The ability of light to activate some phytochemicals may further extend such a synergism during retinal degeneration. In this way, photosensitive natural compounds may produce light-dependent beneficial antioxidant effects in AMD.

## 1. Introduction

### 1.1. Outline of the Main Objectives

The present manuscript aims to highlight how natural stimuli such as specific long-wavelength (amber, red, infrared) light exposure and naturally occurring plant-derived agents (phytochemicals, such as resveratrol, lutein, bilberry and curcumin) may serve as powerful remedies in the course of age-related macular degeneration (AMD). The propensity of outer retinal cells to degenerate in the course of AMD is related to the large number of reactive oxidative species (ROS) which impact the photoreceptors and the retinal pigment epithelium (RPE) mostly at the macular level, where high visual acuity and detailed visual skills are achieved. In fact, in this area, small cones are present, which are activated by light stimuli owing to blue wavelengths, which produce the highest amount of oxidative damage. This is also sustained by a strong oxygen demand by these foveal cones. Thus, high oxygen supply occurs in the macular region of the retina, which is joined with strong exposure to ultraviolet (UV) as well as white and blue light. All these factors lead to a unique number of ROS, which foster the onset and sustain the course of AMD. At present such a disorder lacks an effective cure, and therapeutic efforts often fail or lead to unbearable side effects. Therefore, it is tempting to analyze the beneficial effects of natural stimuli in counteracting the damage induced by ROS and promoting the recovery of visual function. Within this frame, a special emphasis is given to analyze the innumerous functions of RPE, and how these are stimulated by specific wavelengths and phytochemicals, mostly involving the recruitment of autophagy. In fact, autophagy is essential in counteracting oxidation and removing the molecular and cellular damage produced by oxidative stress acting on a variety of molecules and cell organelles. Autophagy is important also to provide biochemical support of retinal visual function and maintaining retinal integrity. In fact, even in baseline conditions, the outer retina including the RPE possesses a high autophagy rate to counteract the large number of oxidative species and support phototransduction. This activity is exacerbated in the course of AMD, when the oxidative damage is increased. Thus, empowering autophagy in the outer retina through natural stimuli may be a promising approach to counteract the damage occurring in AMD. This manuscript aims to dissect how naturally occurring long-wavelength lights and phytochemicals per se may serve as antioxidants and activate autophagy. A further aim of this manuscript consists in showing how the combination of these specific lights and phytochemicals may create a synergism in counteracting AMD-related oxidative damage and stimulating retinal autophagy. In addition, the potential preconditioning of short pulses of blue oxidizing light is considered to induce compensatory mechanisms to prevent the damage induced by oxidative stress.

### 1.2. The Main Functions of Retinal Pigment Epithelium and a Target of Reactive Oxygen Species (ROS) in the Course of Retinal Degeneration

**Figure 1:**
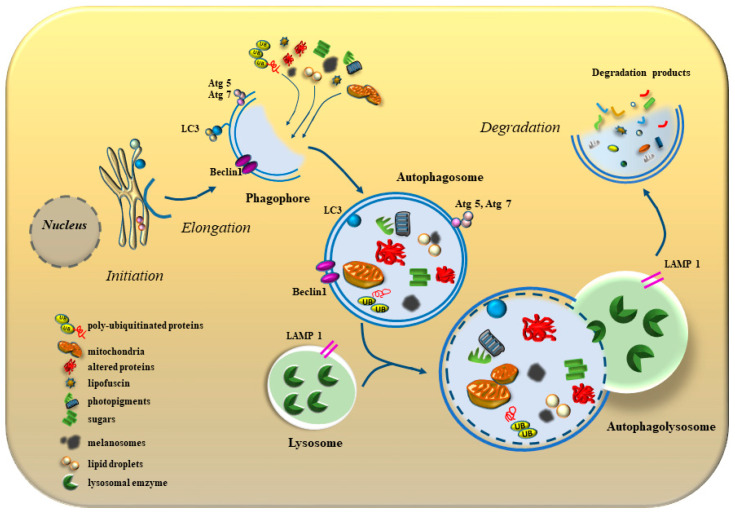
Diagram showing the main autophagy steps. During baseline conditions and mostly upon oxidative stress, RPE features a large number of autophagy-related proteins such as LC3, Atg5, Atg7 and Beclin1. These are produced to commit vesicles from the trans-Golgi network to initiate and elongate the early autophagy structure named phagophore. At this level, specific substrates, including poly-ubiquitinated proteins, mitochondria, lipid droplets, melanosomes, lipofuscin and sugars, start to accumulate within RPE, where they are expected to be degraded. Substrate accumulation increases when the vesicle ceils to form an autophagosome, which further merges with lysosome to constitute an autophagolysosome, where the clearance of oxidized aged components is completed to produce a number of degradation products.

Age-related macular degeneration (AMD) is the most prevalent retinal degenerative disorder leading to blindness, which typically involves the macular region of the retina. The disorder is clinically evident as a loss of visual acuity [1] and the presence of visual distortion (metamorphopsia) [1,2,3]. At early stages, visual impairment is restricted to specific skills such as reading text and facial recognition [4]. In fact, the disease mostly impairs those photoreceptors placed in the macula, which provide the highest visual discrimination and feed the highest cortical integration [2,4]. This is why, at early stages, AMD involves those photoreceptors projecting to the primary visual cortex that carry information to be delivered within the cortical ventral stream [5], thus providing the inputs which allow facial recognition. In other words, AMD at onset recruits the most vulnerable photoreceptors, which correspond to those implicated in the highest level of visual information and integration within supra-modal cortical areas [6]. This is not surprising, since AMD mostly affects small cones in the macular region, which are activated by light stimuli rich in white and blue wavelengths, which are those producing the highest amount of oxidative damage [7,8,9,10]. This mostly occurs in the RPE as well as within outer segment of cones placed in the external retina, which are included between the cell processes of the RPE. In fact, outer retina and mostly RPE are primarily affected in the course of AMD [3,4,11,12]. This is mainly due to the physiological role of RPE, which is constantly engaged by a large number of reactive oxygen species (ROS) produced during direct exposure to natural light in the macula. In fact, within RPE, direct light stimulation impacts the structures owing to a high rate of oxidative metabolism [13]. This increases within RPE during the cycle of light stimulation, which generates a strong amount of energy, which is spent to depolarize photoreceptors and convert light-sensitive molecular species. These phenomena add on the neural properties of the retina and magnify the oxygen demand mostly in the outer retina at the level of RPE. This generates a site-specific pro-oxidant environment, which partly explains why RPE is so prone to early degeneration in AMD. The high oxidative metabolic activity of RPE, beyond producing oxidized species and organelles, is fundamental to recycle the outer segment of the photoreceptors, buffering glutamate and reducing retinoic acid into 11-cis-retinal [3,14,15]. Therefore, in order to counteract such a large number of ROS, RPE integrity strongly depends on intense autophagy activity even in baseline conditions in healthy subjects [16,17,18,19,20] (Figure 1).

**Figure 2:**
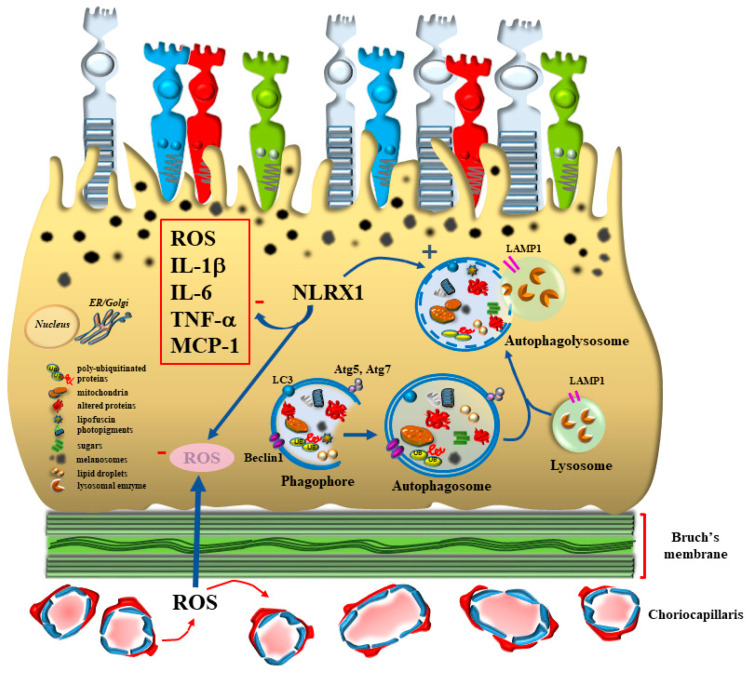
The pro-autophagy effects of retinal NLXR1. Within RPE cells, when ROS increase, a specific compound named nucleotide-binding oligomerization domain (NOD)-like receptor X1 (NLRX1) is produced. This is a powerful autophagy inducer, which counteracts retinal oxidative damage and inflammation. In fact, NLRX1 counteracts ROS formation and ROS-mediated pro-inflammatory compounds such as IL-1β, TNF-α, IL-6 and MCP-1. These occur along with NLRX1 autophagy activation, which fuels the autophagosomes and accelerates autophagy progression into effective lysosomes. Overexpression of NLRX1 reverts inflammasome activation.

This explains why, even in healthy subjects, and in baseline conditions, the autophagy flux within RPE is consistently higher compared with inner retinal layers [12,21]. This allows autophagy within RPE cells to maintain homeostasis, sustaining vision and counteracting oxidative stress. A number of mechanisms and stimuli are powerful autophagy inducers; the intense research in the field of retinal metabolism provides increasing evidence of multiple facets of retinal autophagy beyond counteracting retinal oxidation. For instance, in a recent study, Wang et al. [4] demonstrated that RPE cells produce nucleotide-binding oligomerization domain (NOD)-like receptor X1 (NLRX1), an autophagy inducer, which counteracts retinal oxidative damage and inflammation. In fact, ROS trigger the formation of pro-inflammatory compounds in the retina such as interleukin 1beta (IL-1β), tumor necrosis factor alfa (TNF-α), IL-6 and macrophage proteine-1 (MCP-1) [4] (Figure 2). These chemical species are detrimental for the autophagy pathway as shown by increased p62 levels and decreased microtubule-associated protein 1A/1B light chain 3 (LC3)II/LC3I ratio. Overexpression of NLRX1 within RPE cells reverts autophagy inhibition and suppresses the levels of ROS and inflammasome [4].

In contrast, knocking down NLXR1 inhibits autophagy and activates inflammasome, which sustains AMD [4]. The newly discovered activity of NLRX1 as an autophagy inducer in RPE cells adds on a number of pro-autophagy stimuli within RPE cells, which occur after light-induced modulation of autophagy genes and include ezrin [21], Atg 5 [22], LC3 [23] and at least 23 autophagy-linked genes such as Bcl-2-associated X protein (Bax), forkhead box O3 (FOXO3) and mitogen-activated protein kinase (MAPK)-dependent signaling pathway [24]. Innumerous proteins related to autophagy are produced by RPE cells, including beclin1, PINK1, Parkin and other proteins involved in mitophagy and mitochondrial turnover. All these proteins accompany the high expression of autophagy-related organelles such as lysosomes and autophagosomes, which occur in the greatest number within RPE compared with all retinal layers. This makes the RPE the autophagy-competent retinal layer, which drives the clearance of a plethora of oxidative-dependent toxic substrates.

In fact, RPE cells possess a powerful antioxidant machinery making it possible to counteract retinal injuries and to serve a number of physiological functions, which are required to sustain vision and retinal integrity. In fact, RPE cells are key in providing melanin synthesis [20,25] and handling free radicals produced by intense white/blue light exposure [26,27,28]. Similarly, RPE cells are key in neutralizing ROS produced by signal transduction and phototransduction [29,30]. RPE cells are needed to reduce retinoic acid into its active aldehyde 11-cis-retinal. RPE represents the cell compartment, which recycles damaged outer segments of photoreceptors, where the opsin-packed disks are deranged by the oxidizing effects of light [31,32,33]. RPE cells operate a sort of autophagy of photoreceptor discs through LC3-associated phagocytosis (LAP) [23]. Moreover, RPE cell activity is key in neutralizing ROS, even when these species reach the retina from the choroid through the blood stream [29]. Independently from their origin (within the retina or systemic, through the blood stream), ROS promote both oxidation of lipids, nucleic acids, sugar and proteins and formation of misfolded molecules which need to be quickly removed [34,35]. Similarly, ROS alter mitochondrial activity and disrupt fine mitochondrial structure, which in turn may generate an additional number of ROS [36]. Thus, the quick removal of altered mitochondria and the genesis of novel healthy mitochondria are key to promote the survival of RPE cells in oxidizing conditions. The autophagy machinery is able to subserve all these functions, since it degrades misfolded proteins and oxidizes lipids (lipophagy) [37,38,39] while removing altered mitochondria (mitophagy) and promoting mitochondriogenesis [40,41].

### 1.3. The RPE from Melanin Accumulation to Formation of Oxygen-Dependent Inclusions (Figure 3)

RPE cells develop from the outer membrane of the optic calyx, which expands from the diencephalic vesicle [42]. These cells stay steady during development as a mono-layer of melanin-containing neural cells [43,44]. The synthesis of melanin is key for the activity of the RPE cells, since it allows the absorption of the light, which otherwise would spread between photoreceptors dispersing visual discrimination and causing an excess of oxidation. At the same time, melanin-rich inclusions, named melanosomes, which are widespread within RPE cell processes, suppress the effects of ROS produced by phototransduction and absorb these compounds, thus preventing oxidative damage [27,45]. The production of melanin is strongly regulated by norepinephrine (NE)-containing, tyrosine-hydroxylase (TH)-positive axons [20,25,46]. The presence of melanin within RPE cells is a powerful regulator of cell shape and phenotype [47]. In fact, when comparing albino and pigmented retinas in rodents, there is a marked difference concerning RPE cell size, shape and structure [20,47]. In fact, in the absence of melanin, RPE cells are flat, small, and they lack distal processes, as it occurs in albino eyes, where RPE cell processes do not embrace the distal segment of photoreceptors [47]. The thickness of pigmented RPE is related to melanin bodies, which fill the cells and push the pigment to fill the cell processes placed between the outer segments of photoreceptors [20]. Thus, TH-positive sympathetic innervation of RPE, by increasing melanin content, modulates cell volume and phenotype, including cell biochemistry [48]. The presence of melanin within RPE cells prevents the detrimental effects due to an excess of white light stimulation, which otherwise would produce an excess of toxic ROS and free radicals [27,45].

**Figure 3:**
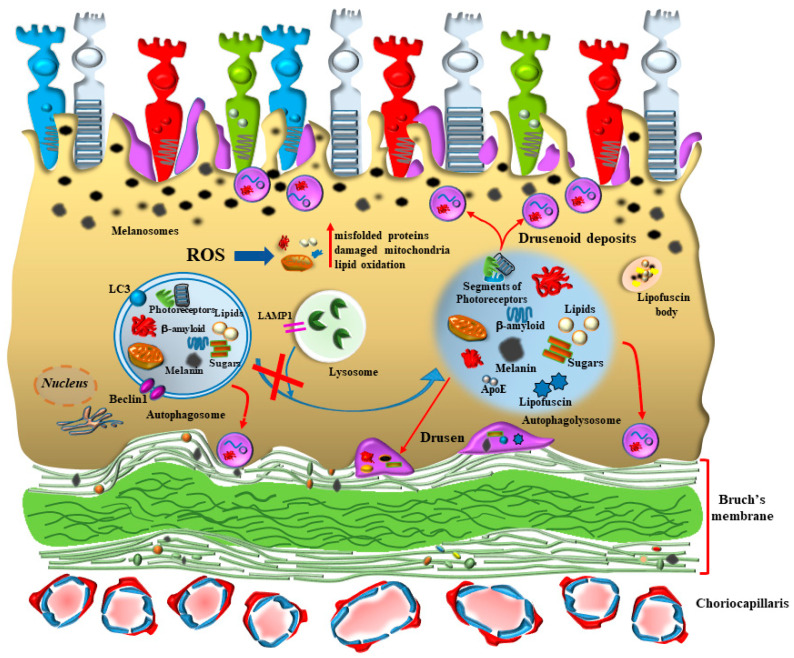
Retinal degeneration following a severe oxidative stress. Upon white light exposure, and following the diffusion through the choroid, a number of oxidant effects occurs within RPE. This is enabled by a high level of oxidative metabolism, which is needed in baseline conditions by RPE cells in order to carry out a number of functions. When ROS increase above a critical level, a number of proteins, lipids, nucleic acids and sugars as well as mitochondria are structurally altered and may produce toxicity and become difficult to be digested. In this way, an engulfment of autophagosomes and autophagolysosomes takes place. The latter are recognized since they contain lysosome-associated membrane glycoprotein 1 (LAMP1). This may lead to the occurrence of giant dysfunctional LAMP1-positive autophagolysosomes within RPE cells as an early pathological finding in the course of AMD. Concomitantly, these non-digested structures and non-cleared organelles may take alternative routes to be extruded from RPE cells. These unconventional secretions may lead to extracellular aggregates named drusen between RPE cells and Bruch’s membrane, or sometimes extracellular aggregates may be transferred towards the opposite domain of RPE approaching the outer segment of photoreceptors as drusenoid deposits.

In addition, melanin binds to lipids and sugars, thus trapping advanced glycation end products (AGEs), which occur in large numbers during AMD, within intra- and extracellular inclusions [49,50,51]. In this way, melanin may work as a sort of buffer trapping various toxic chemical species and forming inclusions, which provide a neutralizing effects against cell damage. In fact, melanin takes part in the inclusions, which are produced during oxidative stress, being observed within drusen or other retinal aggregates, which accumulate in the space between RPE and Bruch’s membrane (Figure 3). As a side note, within this context, it is important to specify that the occurrence of retinal inclusions, including drusen and other RPE aggregates does not necessarily represent a detrimental factor. In fact, they may simply evidence the occurrence of a compensatory segregation of toxic species, which otherwise may be more deleterious for cell survival. Therefore, the significance of retinal inclusions for the fate of the cells during AMD remains debatable, and their protective role is likely to take place (Figure 3). This concept is reminiscent of what is already postulated about the occurrence of neuronal inclusions in the course of degenerative disorders within central nervous system (CNS) [52,53]. In line with this, Herrera et al. [54] claim that such inclusions in the course of retinal degeneration are of inert or even protective nature. The authors of this work express a concept which assimilates retinal inclusions to neuronal inclusions in the course of CNS degenerative disorders [53,55]. In fact, the authors of this manuscript show that melanin accumulation is beneficial to counteract cell damage in the course of retinal degeneration [54]. This is in line with the property of melanin to trap toxic species in the form of inert aggregates, thus occluding their toxic effects. In this way, the occurrence of aggregates and inclusions within RPE cells, which happens often during AMD, may be viewed as one of the protective mechanisms generated by RPE cells to maintain retinal integrity.

## 2. The Specific Role of RPE beyond Baseline Conditions at Onset and during AMD

The presence of RPE is key to produce trophic effects. This is based on the synthesis of compounds and organelles by RPE cells and through the delivery of nutrients coming from the inner choroid towards photoreceptors [56]. In this way, a selective uptake of compounds essential for retinal integrity contributes to the beneficial effects exerted by RPE on the outer retina including the survival of photoreceptors. Nonetheless, most of these effects remain confined to the specific biochemistry of RPE cells. In fact, the activity of RPE is also important for removing iron accumulation, which is associated with oxidative damage in AMD. Exogenous oxidative stress leads to ferroptosis, which consists in Fe^2+^ accumulation and lipid peroxidation in RPE cells [57,58,59]. RPE cells own a specific gamma-glutamylcyclotransferase-1, an enzyme belonging to the unfolded protein response (UPR), which counteracts ferroptosis [59].

The physiology of RPE cells allows the degradation of a number of oxidized substrates. When this activity is impaired, degeneration may occur. This seems to be the case of AMD. Apart from losing their beneficial effects, in the course of AMD, RPE cells may become the source of pathology for the downstream retina. In fact, when studying AMD-patient-specific RPE cells, the lack of appropriate degradation leads to substrates accumulation and abnormal exocytosis [20,60]. In detail, AMD-RPE possesses enhanced polarized secretion of endovesicles (EVs). These EVs carry toxic, non-digested RNA strands, proteins and lipids, which mediate key AMD features including oxidative stress, cytoskeletal dysfunction, angiogenesis and may transmit between neighboring cells or being deposited between RPE and Bruch’s membrane. The dynamics of EVs may also reach the opposite domain of RPE cells to place between RPE and photoreceptors. It is remarkable that EVs derived from AMD-RPE may induce aggregation of amyloid fibrils and drusen formation. In fact, as reported by Kurzawa-Akanbi et al. [61], when control RPE is exposed to AMD-RPE, apical EVs may shift the phenotype of control RPE towards AMD-RPE. In fact, control RPE exposed to AMD-derived EVs develops stress vacuoles, cytoskeletal destabilization and morphological abnormalities.

All these effects indicate how crucial the metabolic activity of RPE cells is, which may sustain retinal integrity or foster retinal degeneration, which mostly depends on neutralizing or increasing the effects of oxidative stress [62,63] through an effective or defective autophagy machinery, respectively [63].

As reported, an excess of oxidative species may alter protein structure and mitochondrial integrity. This constantly occurs in the course of AMD, along with alterations of other chemical species such as lipids, nucleic acids and sugars. In fact, during AMD, oxidative stress is considered to be a major contributing factor [62]. In detail, oxidative stress in the RPE is mainly attributed to an excess of H_2_O_2_, which inhibits mitochondrial turnover and mitochondrial function by suppressing mitochondrial fission and the biogenesis of mitochondria [62]. In fact, H_2_O_2_ alters mitochondrial membrane potential and promotes a vicious circle leading the stagnant altered mitochondria to generate an additional number of ROS.

### 2.1. The Seminal Role of Autophagy within AMD-RPE

The RPE-derived ROS are toxic to a number of molecules such as proteins and lipids by generating abnormal chemical species, which mediate cell damage. In fact, oxidative injury leads to DNA damage and chronic inflammation [13,16,64,65]. Such an accumulation of altered sugars, lipids, proteins and organelles requires powerful systems to degrade and clear these compounds from the retina. This is why when appropriate clearance takes place in the RPE, this impedes accumulation of proteinaceous material and lipofuscins, thus preventing cellular polymorphic debris in the form of drusen [66,67,68]. This requires the occurrence of powerful clearing systems within RPE cells to counteract the innumerous detrimental effects caused by oxidative species. These pathways include the chaperon systems, the proteasome system, antioxidant compounds and mostly the autophagy pathway. Among these, autophagy prevails at large within RPE cells, where it removes oxidized proteins and lipids to counteract AMD [3,16,63]. The proof of principle for such an assumption is provided by the fact that when autophagy is defective, accumulation of misfolded proteins lipids and even specific organelles, mostly mitochondria, takes place leading to experimental AMD [16,17,18,19]. In these conditions, when autophagy cannot be properly activated, or its progression is impeded, misfolded proteins aggregate with sugars within stagnant lysosomes, where they form advanced glycation end-products (AGEs). In these aggregates the occurrence of lipids is abundant to constitute lipofuscins. The effects of noneffective autophagy can be detected both within RPE cells and dispersed in the intercellular space. In fact, the extracellular release of toxic species may occur from RPE cells to spread the disease process during AMD [69]. Among these pathological released species, cell debris and a number of damaged mitochondria are present. Consistently, when autophagy is suppressed, this deleterious release occurs in excess, featuring incompetent lysosomes, misfolded proteins and outer segments of photoreceptors, which are no longer cleared by defective autophagy [70].

### 2.2. RPE Autophagy Defect and Inclusions

Within neurodegenerative disorders, the nature of inclusions represents a hot topic in the whole field of neuropathology [71]. Current vistas are now showing that these consist of mixed aggregates, where proteins lipids and sugars participate in variable numbers along with membranous organelles. In fact, within inclusions, the mass generated by organelle membranes prevails. Most of these organelles belong to a defective autophagy-endo-lysosomal pathways. Such a structure of inclusions is now extending to the retina and specifically to inclusions occurring within or close to RPE cells during AMD. This confirms the relevance of an autophagy defect, which produces accumulation of various oxidized chemical species along with dysfunctional organelles, which otherwise are removed by the autophagy machinery. In line with the polymorphous nature of inclusions in AMD, Hyttinen et al. [63] reported that within AMD, drusen contain lipofuscin and melanosomes which may overlap within single structures (lipomelanofuscin) [72]. This lipomelanofuscin may mix with a number of autophagy substrates and photopigment residues. During AMD, the lack of an effective autophagy produces at first the intracellular accumulation of autophagy substrates, later on non-digested structures move from dysfunctional RPE cells to be released in the extracellular space. In fact, during early stages of AMD, when extracellular drusen are not evident yet, their specific components can be already detected as aggregates within RPE cells, mostly being placed within inert lysosomes [66,73,74] (Figure 3). These findings strengthen the relevance of a defective autophagy in AMD, which is no longer capable of digesting toxic species formed within RPE cells and release these compounds in the extracellular space following a transient intracellular accumulation. In fact, when an autophagy defect occurs, this is quickly sensed by RPE cells which suddenly develop a large number of oxidized autophagy substrates. Later on, these structures are release extracellularly, where they may transmit to neighboring retina. It is likely that the toxicity of this material is lower when being segregated within extracellular inclusions than when being present as toxic chemicals within the cell. In fact, the effect produced by these aggregates is likely not to play a relevant role in AMD, compared with the toxicity exerted by cytosolic-free, oxidized molecules or damaged mitochondria, when they are not yet agglomerated within extracellular inclusions. Thus, as reported in the previous paragraph, inclusions would represent a strategy to trap toxic species to convert them into inert aggregates. In line with this, AMD symptoms seem to be more the consequence of an altered metabolism produced by oxidized structures rather than the mechanical impairment induced by inclusions or aggregates. This is consistent with the hypothesis that a biochemical defect, which is caused by a defective autophagy, is more relevant to the impairment of the visual processing rather than the deleterious effects on vision produced by inclusions or aggregates.

### 2.3. RPE Autophagy Defect and Visual Function

In this way, the role of impaired autophagy becomes relevant compared with the role of an accumulation of autophagy substrates. According to this hypothesis, the loss of visual acuity may be driven by an upstream biochemical dysfunction of cell clearance. Thus, measuring the loss of the autophagy status would predict the severity of AMD to produce visual symptoms more than the amount of the drusenoid area [2]. In fact, a biochemical alteration, which decreases the clearance of oxidizing species, would be directly responsible for the loss of visual acuity while being secondarily related to morphological alterations such as aggregates represented by drusen and loss of planar retinal arrangement. This hypothesis is confirmed by a recent study [21] showing that RPE cells produce autophagy-dependent genes, which need to be activated according to a specific timing and spacing. The timing varies depending on the specific nature of these genes and their function. These genes are responsible for key functions of RPE cells such as the digestion and recycling of intracellular and photoreceptor-derived components. The regulation of these genes requires quick changes in response to daily light and stress conditions. This is key for autophagy to finely tune protein expression and maintaining visual processing. This is essential to counteract visual impairment in patients affected by AMD [21]. Thus, the requirement of an effective autophagy within RPE needs to match the ROS caused by light exposure as well as focal and systemic light-independent excess of oxidative species. An alteration of such a delicate orchestration impairs vision in real time while progressively producing delayed extracellular deposits, which may be not directly involved in producing visual deterioration. This explains why autophagy stimulation may suddenly improve visual acuity due to a direct effect on retinal metabolism [75]. Among key proteins sustaining vision and depending on autophagy activity, RPE produces the so-called retinal-pigment-epithelium-derived factor (RPEDF), which restores the visual cycle leaving intact retinal aggregates. In order to keep steady visual acuity, fast metabolic changes need to be produced, which are compatible with sudden biochemical effects sustained by autophagy activation within RPE cells. These requirements correspond to the activity of autophagy-dependent steps within RPE as indicated by Datta et al. [76]. These include the fast phagocytosis of the outer segment of photoreceptors and counteracting the uniquely high photo-oxidative stress. In line with this, the photosensitive recycling of visual pigment depends on the autophagy status as well as the fast autophagy-dependent receptor turnover in photosensitive neurons. The efficacy of these steps governed by ongoing autophagy which require a few msec within the RPE cells is quick to sustain vision at the retinal level. The pathology of retinal degeneration is still autophagy-dependent and evidences a noneffective antioxidant response, but this pathology develops slowly and accumulates over time. In line with this, we recently published that an impairment of vision during AMD does not necessarily depend on the amount of drusen. In fact, as reported in Pinelli et al., 2020 [2], and in Table 1, which adds additional 18 AMD patients, a discrepancy can be often detected in AMD patients between the amount of the drusenoid area and the loss of visual acuity. The fast timing of autophagy acting within RPE to sustain vision is further proven by data showing the effects of pulsatile light exposure [12]. In these conditions, the stimulating effects of light on autophagy structures are quickly produced according to time intervals from a few seconds up to a few minutes. This occurs as the consequence of light exposure. In fact, during periods of light, autophagy-dependent molecules are upregulated, and more autophagy structures such as autophagosomes and lysosomes are generated. This cycle of autophagy activation is very quick, and its variations occur innumerous times in a day, being suddenly induced by light exposure or suppressed by light deprivation. This sort of ultra-circadian regulation consists of light-induced cyclic variation of autophagy occurring many times during the day, which depends upon direct light stimulation [77]. In fact, autophagy is induced by the process of phototransduction, when a number (at least 23) autophagy-related genes are activated [24]. Some of these genes provide a fast progression of autophagosomes to merge with lysosomes, which is a key step in sustaining vision [78]. This explains the massive number of autophagosomes and active lysosomes which occur in the retina and mostly within RPE, following light exposure. [60,63,78,79,80,81]. Other genes are related to diverse visual steps. In fact, RPE cells recycle the outer segment of photoreceptors by phagocytosis [14,15,82]. This process is related to autophagy since it occurs through LC3, and it is named the LC3-associated phagocytosis (LAP) [23]. In this process, the phagocytic vacuoles containing the outer segment of both rods and cones recruit LC3, thus generating a sort of photoreceptor-dedicated autophagosomes, which quickly digest the disk membranes, which are the photosensitive domain of photoreceptors. In this way, LAP represents a sort of dedicated autophagy/phagocytosis which is crucial for sustaining the physiology of vision at the biochemical level. It is likely that when autophagy is impaired a loss of visual acuity takes place before that a loss of integrity of photoreceptors is detectable and long before drusen are visible in the retina. In this way an autophagy failure, at early steps of AMD, would solely manifest as a loss of visual acuity mostly evident for visual discrimination. This would be caused in part by an impairment of the physiological turnover of photoreceptor discs due to a defective LAP. The biochemical steps of LAP are regulated by a number of molecules and quickly-inducible genes within the retinal pigment epithelium (RPE) such as melanoregulin [83], rubicon and epidermal growth factor receptor (EGFR) [84].

Other light-induced genes involved in this process are Bcl-2-associated X protein (Bax), forkhead box O3 (FOXO3) and the mitogen-activated protein kinase (MAPK)-dependent signaling pathway.

Table 1 reports the original data concerning visual symptoms, retinal thickness and drusenoid area obtained from 18 patients. Visual symptoms are reported as best-corrected visual acuity (BCVA) and contrast sensitivity. In particular, BCVA was measured by the Jaeger Chart test (near BCVA) and the Snellen Chart test (far BCVA), while contrast sensitivity was assessed by the Pelli-Robson Chart test. The central retinal thickness was measured by retinal topography. Metamorphopsia (detected in Amsler test) represents the distortion in the perception of linear images; it was measured as the degree of loss of linear alignment within horizontal and/or vertical axis. Finally, the drusenoid area represents the quantitative assessment of macular area filled with drusen; it was calculated by multiplying the drusen number by each drusen area (mean diameter of drusen in each retina). As demonstrated in a previous paper [2], the drusenoid area is more accurate than drusen number or drusen size to express quantitatively the amount of the macular region filled with drusen. The central thickness obtained at retinal topography provides an indirect measurement of the loss of planar arrangement of the retina. For each of these 18 patients, a representative optical coherence tomography (OCT) is reported in Supplementary Figure S1.

## 3. Natural Light Stimulation and Photobiomodulation (Figure 4)

In line with these findings, Intartaglia et al. [12] postulate that pulsatile induction of autophagy improves therapeutic strategies to treat devastating retinal disorders. The needs of an effective autophagy in the process of vision are in line with the stimulating effects of light on autophagy structures. These concepts assume a practical relevance in the process termed photobiomodulation (PBM), which refers to the natural light stimulation of biological targets. Photobiomodulation may be carried out by delivering short pulses of light owing to various wavelengths. The time duration which is based on short periods exerts a stimulatory effect on the autophagy machinery. In fact, various ranges of wavelengths stimulate a number of cellular functions via activation of photoacceptors [85,86,87].

**Figure 4:**
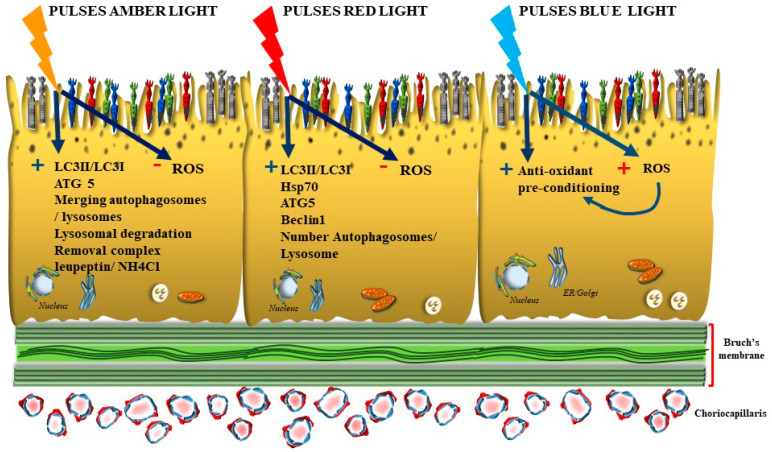
The effects of various wavelengths on autophagy and ROS within RPE cells. Amber light and red light possess a direct antioxidant effect. This is magnified by counteracting the deleterious effects of ROS via activation of the autophagy machinery. In fact, these wavelengths activate multiple autophagy steps, including lipidation of LC3I, increasing the expression of Atg5. The increase in *ATG5* is paralleled by the increase in Atg5 protein. These effects extend downstream, promoting the autophagy flux by merging autophagosomes with lysosomes and later on by promoting lysosomal degradation. Such a physical-chemical interaction is also achieved through the interaction of light with the complex leupeptin/NH_4_Cl, which inhibits lysosomal activity; red and amber light remove the complex from lysosomes, thus allowing quick autophagy activation. In this way, long wavelengths produce sudden effects fostering autophagy-dependent improvement of visual acuity. Later on, this is effective in clearing those substrates which accumulate in the course of retinal degeneration. In contrast, prolonged exposure to blue light produces a direct increase in ROS within RPE cells and inhibits autophagy. However, when short-lasting pulses of blue light are delivered, a compensatory increase in autophagy proteins may take place with the aim to mitigate the damage induced by ROS production (light-induced retinal preconditioning, see text).

It is fascinating that the effects of these wavelengths converge on cellular targets, which are a part of the autophagy machinery. For instance, amber light spanning around a wavelength of 590 nm activates multiple autophagy steps and increases autophagy-related proteins. In fact, amber light quickly (msec) promotes the lipidation of LC3I into LC3II, which represents a protein starting the autophagy flux. In keeping with early autophagy steps, amber light increases the expression of Atg5, which is an early autophagy inducer. The ATG5 gene is part of the 23 autophagy genes, which are increased at different time intervals depending on the specific function, upon light exposure. The increase in ATG5 is paralleled by the increase in Atg5 protein. The effects of amber light extend downstream along specific autophagy steps. In fact, amber light promotes the autophagy flux by merging autophagosomes with lysosomes and later on by promoting lysosomal degradation. Such a physical-chemical interaction is achieved through the interaction of amber light with the complex leupeptin/NH_4_Cl. Such a complex exerts a tonic inhibition on lysosomal activity, which is removed by the interaction with amber light. This allows a fast autophagy activation as the consequence of pulses of amber light. This produces sudden effects promoted by autophagy and allows the clearing of those substrates, which accumulate in the course of retinal degeneration [22]. In keeping with long wavelengths, the exposure to pure red light exerts a powerful antioxidant effect, per se, and through the activation of the inducible isoform of the chaperone protein heat shock protein 70 (HSP 70). In addition, red light activates autophagy and removes specific autophagy substrates such as the misfolded isoform of tau protein. In fact, the autophagy proteins LC3 and beclin1 suddenly increase following red light exposure [88]. If one goes back in the autophagy process, similar to that shown for amber light, pure red light increases the primary transcript of early autophagy genes such as ATG5. Such a sudden activation of autophagy also produces a number of late morphological correlates at the subcellular level. In fact, the increase in autophagy-related proteins induced by red and amber light can be visualized by transmission electron microscopy, where the increase in autophagosomes and lysosomes is evident [89,90].

### Oxidative Preconditioning, the Paradoxical Benefit of Quick Pulses of Blue Short Wavelengths Light

If red and amber lights are powerful antioxidant and autophagy inducers, what about the beneficial effects of pulses of blue light exposure? The long exposure to blue light is known to produce detrimental effects on the retina and sustain AMD when applied continuously for considerable time intervals. These effects are based on a high production of ROS and free radicals. However the exposure to blue light in pulses may be beneficial. This puzzling evidence owns a potential explanation within the scenario of preconditioning effects. In fact, it is known that the outcome of a critical ischemia may be improved by ischemic preconditioning, which occurs through previous compensatory activation of autophagy [91,92]. In this way, one may hypothesize that short exposure to blue/cyanide light when repeated in various quick cycles may activate antioxidant mechanisms by upregulating specific proteins as a compensatory attempt which would provide a protective proteomic background to prevent the onset and the progression of AMD. In line with this hypothesis, the light-induced increase in autophagy genes following pulses of light may also work as a compensatory adaptation, which ends up to elevate the retinal resilience to oxidative conditions.

In fact, prolonged exposure to blue light is detrimental for the retina, and it is claimed to act as the main promoter of macular degeneration. When blue light is delivered for prolonged time intervals, it produces an excess of oxidative stress, and it is deleterious in AMD. The toxicity of prolonged blue light exposure affects all retinal layers. At the level of RPE, this causes altered mitochondria and a severe oxidative stress, which is produced by the light per se and by altered mitochondrial metabolism [93]. In fact, filtering blue light protects mitochondria and promotes the integrity of RPE cells [94]. The deleterious effects of blue light were recently found to involve miR-27a, a negative regulator of forkhead box O1 (FOXO1). Following prolonged blue light exposure, an upregulation of miR-27a causing a reduction of FOXO1 is documented, and it induces ROS accumulation, oxidative stress and cell death in RPE cells [95].

Therefore, blue-light preconditioning should be aimed at reducing the time of exposure to intervals allowing a proteomic compensatory response without leading to retinal damage. In fact, recent experimental evidence indicates that when blue light is delivered in pulses, a long-term protective effect may occur in the presence of prolonged exposure. In these conditions, a compensatory adaptation occurs when photoreceptors upregulate stress response pathways while reducing the expression of phototransduction components, ion transporters and calcium channels. Such a shift in gene expression promoted by short-lasting blue light ameliorates the oxidative stress induced by long-lasting blue light exposure [96]. Thus, a blue-light antioxidant preconditioning seems to occur also for the oxidizing effects induced in the retina, when these are properly tuned and delivered prior to the oxidizing stimulus. The preconditioning-induced neuroprotective gene pattern may counteract oxidative stress. A sound example of this effect was induced in flies by 3 h daily exposure to blue light, which increased transiently ROS but not enough to induce cell degeneration. After a certain cycle of this ROS preconditioning, flies can tolerate 8 h blue-light exposure without any degenerative change. In mammalian species, the effects of blue light are similar. In fact, although prolonged blue light exposure fosters retinal damage, a short time of exposure may produce compensatory effects, which counteracts subsequent oxidative stress by increasing the tolerance to blue light exposure for prolonged time intervals. This appears to occur mainly through the stimulation of lysosome synthesis. In fact, short-lasting blue light promotes the nuclear transport of transcription factor EB, which in turn increases lysosomal-related gene expression [97]. The timing of short periods of blue light needs to be finely tuned aiming at promoting a compensatory response, through which retinal preconditioning may counteract the damage induced by prolonged blue light exposure. These concepts are in line with recent studies showing the relevance of a stimulated autophagy to sustain protection through post-ischemic conditioning in the ischemic retina [98].

## 4. The Autophagy-Inducing Effects of Phytochemicals

A number of naturally occurring compounds named phytochemical were intensely investigated in recent years to counteract oxidative cell damage. In fact, most of these compounds are powerful antioxidants and autophagy inducers [3,99,100] thus connecting the main mechanisms being involved in AMD. Some of these compounds, apart from inducing a sudden autophagy stimulation, are able to exert an epigenetic control on autophagy related genes [101,102,103,104].

Phytochemicals were challenged at the clinical and experimental level in a number of retinal disorders, including systemic disease affecting retinal integrity such as diabetes [105,106,107]. Due to a powerful antioxidant effect, phytochemicals were suggested to play a role to treat AMD [99]. In fact, most treatments of AMD are focused on plain antioxidant agents and compounds being able to suppress the genesis of new vessels. This is also the case of phytochemicals, which counteract oxidative stress and suppress vascular endothelial growth. This is the case of a number of compounds reviewed by Bosch-Morell et al. [108] such as saffron, ginkgo, bilberry and blueberry, curcuma, carotenoids, polyphenols and vitamins C and E. A clinical study is available showing that in AMD patients, the combined administration of bilberry, lutein and resveratrol repeated daily for several months produce a suppression of the drusenoid area [2,99]. In detail, this consists in clearing extracellular aggregates at the retinal choroid border just between the retina pigment epithelium (RPE) and the Bruch’s membrane. This occurs along with a consistent improvement in visual acuity. Since the pathogenesis of AMD features oxidative stress and abnormal angiogenesis, and both effects are strongly modulated by the autophagy status, a natural question is whether phytochemicals act as autophagy inducers. In fact, in the early part of the manuscript, evidence is provided that a defective autophagy is key in sustaining multiple facets of AMD. In addition, the autophagy machinery besides being responsible for most pathological alterations of AMD is critical to sustain quick biochemical events involved in visual detection. Thus, the ability of phytochemicals to modulate autophagy is relevant to consider potential disease-modifying effects in AMD in keeping with preliminary findings about a protective effects of some nutraceuticals (the combination of lutein, resveratrol and bilberry) in the course of AMD.

The prominent effects of phytochemicals described below are reported in Table 2.

### 4.1. Lutein

It is remarkable that lutein does stimulate autophagy, and it may counteract the deleterious effects induced by autophagy inhibition [113]. Chang et al. [109] tested lutein as an autophagy inducer, as shown by a dose-dependent increase in the intracellular amount of LC3. It is likely that most of the antioxidant effects exerted by lutein are obtained through autophagy activation, since this compound elevates a number of steps in the autophagy machinery, which were extensively reported by Chang et al. [109]: (i) lutein increases the levels of the autophagy inducers LC3 and Beclin1 while (ii) increasing the primary transcript of early autophagy genes such as ATG4A, ATG5, ATG7, ATG12, and (iii) further on in the autophagy pathway, lutein increases the number of autophagosomes; (iv) lutein promotes the progression of the autophagy flux; (vi) the effects of lutein extend to activating the autophagy-related molecules’ AMP-activated protein kinase (AMPK), Jun N-terminal kinase (JNK) and p-38. Among various steps affected in the autophagy pathway, the increased expression of the primary transcript BECN1 and the overexpression of its protein beclin1 appear pivotal in inducing the autophagy machinery, which cannot be stimulated anymore by lutein administration when BECN1 is knocked out [109]. The marked activity of lutein as antioxidant/autophagy inducer is promising when considering the pathophysiology of AMD. Remarkably, when lutein is administered systemically, the compound is concentrated in the retina mostly at the macular level [117,121,122], which is fascinating considering that such a small region is the target of degeneration during AMD. In fact, a recent clinical evidence indicates that lutein and its metabolite zeaxanthin when administered to AMD patients improve the disease status [123].

The lack of ability to synthesize lutein in the human body and the evidence for a therapeutic efficacy of this compound led to the recommendation of a diet rich in lutein and zeaxanthin during AMD [124]. The natural distribution of lutein in the macula joined with the powerful effects of lutein as an antioxidant for the RPE makes this phytochemical a promising compound to be administered chronically in the course of AMD. However, the poor solubility and bioavailability of lutein administered orally fostered the development of soluble and diffusible analogs. In this way, lutein di-glutaric acid, which is a soluble and diffusible lutein pro-drug, was tested for its antioxidant effects within RPE cells [110]. In this study, lutein di-glutaric acid was effective in preventing RPE damage due to H_2_O_2_ and in producing the same activation of biochemical cascades including autophagy proteins described for plain lutein. The powerful antioxidant effects of lutein and its soluble analog are achieved also independently from autophagy activation since lutein increases the levels of direct antioxidant molecules such as the enzymatic antioxidants glutathione peroxidase (GPx) and catalase (CAT) and the non-enzymatic antioxidant glutathione (GSH) [110]. Still, it is questionable whether an increase in endogenous antioxidant species is really a direct effect, since the activation of autophagy/mitophagy and the improvement of mitochondrial turnover induced by lutein, apart from counteracting the consequence of oxidative stress, may induce an increase in antioxidant compounds [111]. In fact, the improved mitochondrial structure increases the level of a number of mitochondrial-derived peptides such as humanin, which increases the level of mitochondrial GSH, and inhibits ROS generation. Similarly, as we reviewed recently, the very same pathway involving autophagy activation in the inner choroid/outer retina, mostly within RPE cells, may produce a powerful anti-inflammatory effect by inhibiting inflammasome and suppressing exudative phenomena and new vessels proliferation, as it occurs in wet AMD [3].

Thus, the beneficial effects of lutein in counteracting oxidative damage in the course of AMD can be placed at multiple steps in the pathophysiology of the disorder. In fact, the efficacy of lutein is achieved in multiple ways, which can be roughly summarized here as (i) promoting a direct antioxidant defense; (ii) activating the autophagy/mitophagy pathway, which neutralizes oxidative species; (iii) activating the clearance of oxidative by-products once they are produced; (iv) removing damaged mitochondria, which become a source of oxidative stress; (v) promoting the synthesis of novel mitochondria; (vi) exerting a powerful anti-inflammatory effect; (vii) inhibiting new vessels proliferation. In addition, lutein as other phytochemicals, may act as an inducer of retinal stem cells, which are stimulated by autophagy activation [2,3,112,120,125,126,127]. Most of these steps are shared by many phytochemicals. In this context, the detailed analysis of lutein is provided as a paradigm to discuss the multimodal activity of phytochemicals to prevent and counteract AMD. Some other compounds will be mentioned due to clinical and experimental evidence, which indicate a beneficial effect in the RPE and AMD.

### 4.2. Resveratrol

Similarly to lutein, resveratrol exerts antioxidant effects on RPE. In fact, when administered to RPE cells, resveratrol inhibits the number of the oxidative by-products such as malondialdehyde, and it increases the catalytic activity of the enzyme superoxide dismutase (SOD) [114]. In keeping with its powerful effects, as autophagy inducer, resveratrol markedly activates autophagy within RPE [113]. As expected, when stimulating autophagy and mitophagy, a concomitant increase in the biogenesis of mitochondria takes place. This occurs through the molecule peroxisome-proliferator-activated receptor-γ coactivator-1 (PGC1)-alpha which was shown to stimulate mitochondriogenesis upon autophagy activation [128]. In fact, following resveratrol an increase in PGC1-alpha occurs [129]. As a consequence of this mitochondrial quality control and function, resveratrol improves mitochondrial activity in the retina [115].

The autophagy-inducing effects of phytochemicals are unlikely to rely on mechanistic target of rapamycin (mTOR) inhibition. In fact, both lutein and resveratrol act as autophagy activators without affecting mTOR activity. The stimulation of autophagy, which occurs downstream to mTOR complex, is visible in RPE cells as increased autophagy vacuoles and increased autophagy flux measured by a progressive augmentation in the LC3II/LC3I ratio, which take place at various time intervals following resveratrol administration, concomitantly with a decreased amount of p62 [130]. Moreover, the merging between autophagosomes and lysosomes is increased following resveratrol [131]. A powerful anti-inflammatory effect is induced by resveratrol either via sirtuin 3 activation [116] or independently, by a direct effect on RPE cells [132]. Such an effect joined with the inhibition of angiogenesis due to the block of vascular endothelial growth factor (VEGF) [133] suggests that resveratrol may be effective both in wet and dry AMD. Similar to the findings reported for lutein, resveratrol may provide beneficial effects on retinal cells due to its properties as a stimulator for stem cell niches as shown in the hippocampus. In detail, this effect which is still mediated by sirtuin consists in committing the stem cell to differentiating towards specific neuronal phenotypes [134].

Despite such an apparent equivalence between the effects described for lutein and those we just reviewed concerning resveratrol, it is important to specify that in the case of lutein all these effects are demonstrated in the retina and within RPE cells. On the other hand, due to a lack of available data, resveratrol was not tested so much at the retinal level and only some of these effects are described within RPE, while others were found to occur in other organs or cell types simply due to a lack of experiments carried out in the retina. This makes resveratrol very similar to lutein as an antioxidant, although the evidence concerning its potential site-specific efficacy within RPE is less documented. In fact, in a PubMed search carried out on 15 February 2023 for the comparison of “resveratrol and retina” with “lutein and retina”, the difference was roughly four-fold.

### 4.3. Bilberry

This site specificity of retinal effects further decreases when considering another promising candidate among phytochemical to be used in protecting RPE, which is bilberry [135]. This polyphenol phytochemical stimulates autophagy [117] and promotes the clearance of misfolded proteins, which accumulate into the drusen, such as beta-amyloid [136] along with lipids, which are similarly cleared by the exposure to bilberry [137].

Most of the effects which are attributed to bilberry depend indeed on the activity of its metabolite protocatechuic acid [117]. The activity of bilberry is mostly evidenced concerning the protection against aggregation of misfolded proteins. In fact, apart from beta-amyloid, bilberry clears the cells from misfolded opsins in the outer retina. In detail, bilberry clears and prevents the aggregation of S-opsin, it promotes the activation of activating transcription factor 4 (ATF4), and it induces the expression of primary transcripts of gene inducing proteins involved in the unfolded protein response [138].

The powerful effects of bilberry in counteracting the detrimental effects of AMD for visual response in the retina were challenged by using electroretinogram by Osada et al. [118] both in photopic and scotopic conditions to recruit cones and rods, respectively, within mouse retina. Exposure to bilberry prevents the suppression of both photopic and scotopic retinal responses following a toxic light exposure. This immediate improvement in visual performance is likely to be induced by a protective effects of bilberry against the oxidative stress produced by light exposure.

Bilberry is effective to protect against ROS-induced cell death while producing a decrease in the number of ROS induced by intense toxic light stimuli. In these conditions bilberry preserves the number of tight junctions between RPE cells, which are lost early in AMD [118]. Bilberry exerts protection in the retina, which occurs along with pro-autophagy, antioxidant and even anti-inflammatory [119] effects. The effects of bilberry are evident also at the level of retinal blood vessels, which preserve their integrity [139].

### 4.4. Curcumin

Curcumin represents a very promising, potentially protective, phytochemical to be administered in the course of AMD [140]. In fact, this compound stimulates autophagy both in baseline conditions and following toxic stimuli. The protective effects of curcumin are demonstrated in various cell types, where curcumin acts as an autophagy inducer [120,141,142,143,144,145,146,147]. When looking at retinal site-specific effects, curcumin counteracts apoptosis and inhibits MAPK-dependent signaling in experimental retinal degeneration [148,149]. The protective effects of curcumin are promising in experimental AMD [150,151,152]. In fact, a remarkable tropism of curcumin towards pathological structures formed during AMD is described [153], where curcumin fluorescence and hyperspectral imaging indicate the accumulation of this compound within retinal amyloid β (Aβ) deposits, which suggests a specific tropism for drusen. A recent study indicates that a metabolite of curcumin, hexa-hydro-curcumin (HHC), produces better retinal protection than curcumin itself [9]. Although each single mechanism responsible for neuroprotection remains to be established, the protective effects of curcumin and HHC in light-induced experimental AMD are grounded on autophagy activation and a direct antioxidant effects [9]. A recent study in searching for potential candidates to treat AMD carried out an in silico analysis to identify the off-label protective effects in AMD of a number of chemicals and drugs approved by the Food and Drug Administration for other indications [154]. In this search, a number of compounds belonging to multiple drug classes were tested. Among these, curcumin emerged as the most effective compound in affecting genes involved in AMD. Such an association was still evident when specific genes associated with various isoforms of AMD were tested. In fact, curcumin is the most significant compound for dry AMD including geographic atrophy and wet AMD, being such an association more evident for the dry than the wet forms [154]. These results suggest a very promising protective effect of curcumin in the course of AMD. In fact, the autophagy-inducing properties of curcumin in various cell types are magnificent compared with other phytochemicals [59,145,146,155,156,157,158,159], and they are similar to those reported for rapamycin [144,160,161,162,163,164,165] with the important difference that curcumin does not carry the burden of most side effects induced by rapamycin. The protective effects of curcumin in restoring autophagy in the RPE are comparable to those described for lutein [113]. Again, a major limitation when using curcumin, as well as other phytochemicals, is due to low bioavailability. For this reason, Muangnoi et al. [166] tried with some success the effects of curcumin esters as pro-drugs. These esters produce better effects when administered systemically compared with simple curcumin. Still, it would be relevant to develop therapeutic strategies to treat retinal disorders by a full increase in curcumin bioavailability, which may be achieved through appropriate pharmaceutical designs. In fact, according to Chang et al. [167], curcumin represents an ideal drug that may effectively restore neuronal functions in AMD-patient-derived RPE cells, rendering this drug an effective therapeutic option to treat macular degeneration induced by oxidative stress.

## 5. The Archaic Nature of Synergism between Natural Light and Phytochemicals May Work as a Disease Modifier in AMD (Figure 5)

When looking at the site specificity of retinal autophagy activation induced by light exposure, it is not surprising that the most impressive effects are measured in the outer retina at the level of the RPE within the macula. This is the area where light directly impacts RPE cells and distal photoreceptors. Considering that the macular region possesses the highest concentration of the autophagy stimulator, phytochemical lutein, a natural question needs to be discussed as follows: are these effects of light on the autophagy machinery purely induced by light exposure, or are they produced by the activation of light-sensitive phytochemicals working both as photoacceptors and autophagy inducers? Moreover, considering both stimuli as effective in stimulating retinal autophagy, are light exposure and phytochemicals synergizing? Specifically, since phytochemicals are often light-sensitive and light-activated, is light exposure making phytochemicals more effective as autophagy inducers?

**Figure 5:**
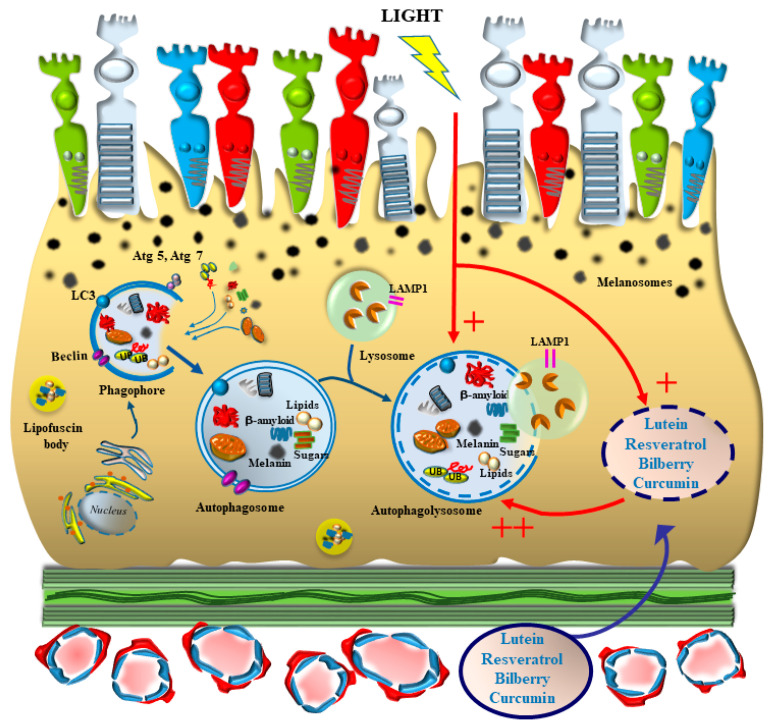
Convergence and mutual enhancement of light and phytochemicals as autophagy inducers within RPE. Specific phytochemicals, which are at present recommended in the diet of AMD patients are strong autophagy inducers. These effects remarkably concern the very same steps which are activated by long light wavelengths (see also Figure 4). In addition, the biochemical activity of phytochemicals is enhanced upon light exposure, since most of these molecules are photopigments which naturally occur in plants to transduce the effects of light to sustain cell viability.

As a matter of fact, when phytochemicals are combined with pulses of amber and red light according to specific timing, the beneficial effects on retinal anatomy and visual acuity are improved [168,169]. In fact, in these manuscripts, common targets are described concerning the effects of amber and red light exposure with the effects produced by phytochemicals.

In these patients, the combination of PBM and phytochemicals produced a marked improvement of visual acuity, which was associated with the clearance of submacular aggregates (drusen). This is in line with the hypothesis that the combination of light and phytochemicals is effective in improving visual acuity and restoring retinal anatomical integrity. These data are also in line with the data provided in a case report previously published [170], in which a patient suffering from AMD improved after PBM with further amelioration following a specific diet with phytochemicals [170]. In detail, in this patient, light exposure produced an improvement of the BCVA (from 20/30 to 20/25) and a reduction of the drusenoid area (from 110,000 mm^2^ to 70,000 mm^2^), which were measured at six months after PBM [170]. Remarkably, the administration of phytochemicals during six months after light exposure markedly enhanced the improvement of both BCVA and drusenoid area. In fact, at 12 months after PBM and phytochemicals, BCVA was completely restored (20/20), and the drusenoid area was further decreased (30,000 mm^2^).

As reported for Table 1, the Jaeger Chart test and the Snellen Chart test were used to measure near and far BCVA, respectively, while the Pelli-Robson Chart test was used to assess contrast sensitivity. Moreover, the central thickness was reported along with the occurrence of metamorphopsia (i.e., the distorted perception of linear images as measured through Amsler test). Finally, the drusenoid area was calculated by multiplying the drusen number by the area of each drusen. Specific patterns of light exposure, when combined with specific phytochemicals, may synergize in improving the microanatomy of the retina by restoring its neurobiology and improving visual acuity in the course of AMD. These effects are evident when comparing data before light exposure and phytochemicals administration (Pre in Table 3) with data obtained after this treatment (Post in Table 3). The specific wavelengths are the following: 590 nm, 660 nm and 850 nm corresponding to amber, red and infrared lights, respectively). These were administered according to a specific timing consisting of a multi-step pattern. The first interval consists of 35 s of amber (590 nm) and infrared (850 nm) wavelengths; the second step consists of 90 s of red (660) wavelength; the third step consists of 35 s of amber (590 nm) and infrared (850 nm) wavelengths; the last step consists of red (660 nm) wavelength applied for 90 s. This four-step pattern is repeated three times a week during a total duration of 1 month. These wavelengths were followed by a diet consisting of 6 g per day of lutein, resveratrol and bilberry which were taken by this patients for 20 days a month, for a total duration of six months. These compounds induce autophagy in the retina (as reported in Table 2).

In these patients, the combination of light according to this timing of wavelengths was carried out in combination with the same diet containing lutein, resveratrol and bilberry as described in the case report which was previously published [170]. These preliminary data show an improvement of visual acuity associated with an improvement of the anatomical integrity of the retina.

It is remarkable that light and phytochemicals induce both autophagy and direct antioxidant molecules. Such a convergence is remarkable when looking at the specific steps in the autophagy machinery. In fact, as reported for light and phytochemicals, both stimuli are strong inducers for early steps in the autophagy machinery by increasing early autophagy genes and proteins such as LC3II, beclin1 and Atg5. In both cases, the number of autophagosomes is increased as well as the merging between autophagosomes and lysosomes. In this way, even the doses of phytochemicals may be decreased (which may allow efficacy with low bioavailability), and the potential preconditioning to counteract oxidative stress in AMD may be carried out more safely. The site specificity of light/phytochemicals convergence is also remarkable, since the retina is the recipient of natural light, and some phytochemicals are reported to accumulate in the retina following systemic administration. It is remarkable that site specificity may further increase when considering specific retinal areas, since lutein is stored within the macula, and the macula is the site where natural light impacts directly the RPE which is the final target to increase autophagy in the course of AMD. At this level, light has direct access to the outer retinal segment at the border with the inner choroid, which is supposed to be the site where phytochemicals may synergize both concerning the RPE and blood vessels of the choriocapillaris. This inner choroid/outer retinal border corresponds to the area where drusen accumulate in the course of retinal degeneration, between RPE and Bruch’s membrane. This is expected to lead to a decrease in the amount of drusen, which is reported in a recent manuscript [168] in a case reporting combined administration of photobiomodulation and nutraceuticals. This is in line with the hypothesis reported in Figure 5.

### Light-Induced Chemical Changes in Phytochemicals

A key question concerning a further step in the synergism between light and phytochemicals relates to the specific structure of most phytochemicals, which are sensitive to light. In other words, how inert phytochemicals are related to the effects of light? May light exposure enhance the autophagy-inducing effects or the natural antioxidant activity of phytochemicals?

As a process which was reported to derive from the evolution of living organelles, since phytochemicals occur in nature mostly in the structure of plants, it is not surprising that these compounds are modified in their structure and function upon exposure to natural light. Similarly, the retina is an anatomical structure, which is developed in the human body under the plastic effects produced by light exposure. Similar to plant surface, the retina possesses molecules, which need to be modified in their structure when impacted by various wavelengths. These facts provide a rationale for convergence of enlightened retina and phytochemicals to trigger similar biochemical pathways. In fact, photoreceptors themselves are modified by light exposure, and melanin is the classic surface molecule, which acts as a photoacceptor in the retina as well as the whole surface of the human body. Thus, it is not surprising that phytochemicals are properly active when exposed to light, which augments the site specificity of the beneficial effects of systemically administered phytochemicals, specifically for retinal structure and function. In this way, it is not surprising that resveratrol is modified upon light exposure [171]. In fact, white light modifies and may enhance the antioxidant properties of a number of phytochemicals [172]. As shown by Morello et al. [173], different wavelengths produce different effects on phytochemicals, and specific wavelengths determine the change in the structure and chemical properties of lutein [174]. This happens also for resveratrol [171]. When a mixture of polyphenols is exposed to light, various metabolites are generated [175].

Therefore, the combination of light and phytochemicals in treating retinal oxidative damage is just repurposing the natural interaction which occurs in nature between light and light-sensitive natural compounds. We may indicate that the interaction between specific wavelengths and specific chemical moieties of phytochemicals represents an evolutionary old interaction, which drove the structure and metabolism of those cells which are exposed to light. It is not surprising that these effects mostly occur within the RPE, which in the retina represents the cell type that absorbs the highest amount of light. The convergence of light and light-sensitive phytochemicals in activating the autophagy machinery is a sort of proof of principle of the significance of such an interaction in the evolution of living species. As shown by Yu et al. [176], in plants, autophagy regulates ROS homeostasis to increase salt tolerance. In plants, similar to the retina, light is essential; however, in plants as well as in the retina, an excess of light generates an excess of ROS which damage plant cells, which is counteracted by increased autophagy within plant cells [177].

Thus, the protective role of autophagy against light-induced oxygen-derived toxic species in the retina recapitulates the same phenomenon which may lead autophagy to prevent cell death in the plants [177]. When autophagy is suppressed in plant cells, ROS-induced damage is enhanced, featuring intracellular alterations which are reminiscent of human AMD such as engulfment of phagophores and big stagnant aggregates of peroxisomes within vacuoles [177]. In plants, phytochemicals are an essential part of the cell structure which is partially lost in the human retina. In fact, plant cells are fully exposed to light. The concept of delivering antioxidant, autophagy-inducing phytochemicals to the RPE under autophagy-inducing light exposure seems to represent a natural way to reproduce the topography of strong antioxidant defense.

The ancestral role of light in inducing cell growth in plant is likely to be conserved in the human retina. In fact, specific wavelengths can stimulate the proliferation and differentiation of retinal stem cells.

## 6. Conclusions

The present manuscript overviews the role of autophagy in retinal degeneration, with a focus on the effects of oxidative stress on RPE cells in AMD. The multi-faceted effects of autophagy in baseline condition and during AMD are mostly evident in the outer retina with an emphasis on RPE cells. At this level, autophagy produces both quick and delayed effects based on metabolic demand, oxidative species and visual transduction.

The stimulation induced by white light exposure requires quick autophagy activation based on rapid enzymatic activity in order to counteract oxidation of chemical species to avoid cell toxicity. However, the quick activation of autophagy is needed also to sustain the process of vision. In fact, the turnover and availability of photoreceptor’s outer segment depend on the quick activation of LC3-associated phagocytosis (LAP). The effects of autophagy on RPE cells involve the activation and synthesis of autophagosomes and lysosomes as well as the progression of autophagosomes to merge with lysosomes. The enzymatic clearance of lysosomal content is also autophagy-dependent as well as the number of lysosomal enzymes. A number of autophagy-related genes are induced by light exposure and modulate a number of autophagy-related steps. This explains why the loss of autophagy is essential in producing the loss of visual acuity and the pathological alterations, which characterize AMD. In fact, the onset and progression of AMD take place in the outer retina, where autophagy activity is more critical and mostly effective even in baseline conditions. The knock out of specific autophagy genes mimics most symptoms and pathology of AMD, while autophagy stimulation may counteract these effects. Despite the fact that exposure to white, blue or UV light is detrimental, as it initiates and worsens AMD, specific long wavelengths in the amber/infrared spectrum may induce beneficial effects. In the present manuscript, a specific analysis of the beneficial effects of specific wavelengths (amber, red and infrared) is reported, and their role in activating autophagy is analyzed. Such a natural and specific light stimulation may be useful to counteract AMD. Similarly, naturally occurring phytochemicals such as lutein, bilberry, resveratrol and curcumin share a remarkable similarity with red/infrared light in stimulating autophagy. In the hypothesis that combined light- and phytochemical-induced autophagy stimulation may be synergic, we reported previous studies and preliminary findings on the effects of such a combination. These preliminary data suggest that combining light (according to specific timing and wavelengths and for specific duration) with the intake of specific doses of some phytochemicals (such as resveratrol, lutein and bilberry) may represent a natural approach to mitigate the course of AMD. Future studies designed with a better tuning of such a combination may provide a sound approach to validation in patients suffering from AMD. Finally, a further approach was considered based on experimental models and patients’ data from ischemic retinal disorders, which consists in oxidative blue-light preconditioning. Although the effects of prolonged exposure to white and blue light are detrimental for retinal integrity and the course of AMD, pulses of these short wavelengths of light, when administered for short time intervals and reiterated a number of times, may lead to refractoriness to retinal damage. In fact, similar to the effects happening during ischemic preconditioning in cardiovascular disorders, the pro-oxidant effects of blue light for short time intervals may induce compensatory changes which are long-lasting and protect the retina from damage induced by prolonged exposure to white light. Preliminary experimental data confirm such hypothesis. Again, further studies designed to better comprehend blue-light preconditioning are needed.

## Figures and Tables

**Table 1:** Visual symptoms and amount of drusen in patients affected by AMD.

Patient	BCVA *	Metamorphopsia	Contrast Sensitivity	Central Thickness (μm)	Drusenoid Area (mm^2^)
1: F, 83 years	20/63	0.4°	2.0	185	7.00
2: M, 74 years	20/80	0.3°	1.7/1.8	182	7.54
3: F, 61 years	20/25	0.3°	1.9	206	2.00
4: F, 85 years	20/25	0.4°	1.9	205	4.52
5: F, 57 years	20/32	0.3°	1.9	183	18.80
6: F, 81 years	20/32	0.4°	1.8/1.9	225	3.14
7: F, 75 years	20/25	0.4°	2.0	200	1.32
8: F, 68 years	20/32	0.5°	1.8	222	11.30
9: F, 81 years	20/32	0.5°	1.9	235	1.32
10: F, 59 years	20/25	0.5°	1.9	238	1.53
11: M, 78 years	20/80	0.3°	1.8	208	13.10
12: F, 72 years	20/25	0.5°	1.9	195	1.76
13: M, 70 years	20/50	0.4°	1.8	212	7.00
14: F, 62 years	20/25	0.5°	1.8	215	4.15
15: F, 78 years	20/25	0.4°	1.8	218	12.50
16: F, 72 years	20/25	0.5°	1.9/1.0	261	3.46
17: F, 72 years	20/32	0.5°	1.8	222	1.13
18: F, 70 years	20/40	0.4°	1.9	211	0.78

* BCVA = best-corrected visual acuity.

**Table 2:** Main effects of some phytochemicals being administered in AMD.

Phytochemicals	Effects	Authors and References
Lutein	activates autophagy	Chang et al., Am J Chin Med, 2017; [109]
	counteracts oxidative stress (ROS)	Muangnoi et al., Int J Mol Sci, 2021; [110]
	increases mitochondrial turnover	Minasyan et al., Oxid Med Cell Longev, 2017; [111]
	exerts anti-inflammatory effects	Pinelli et al., Int J Mol Sci, 2020; [3]
	induces retinal stem cells	Jin et al., Stem Cell Res Ther, 2022; [112]
Resveratrol	activates autophagy	Munia et al., Nutrients, 2020; [113]
	counteracts oxidative stress (ROS)	Yang et al., Eur Rev Med Pharmacol Sci, 2019; [114]
	increases mitochondrial turnover	Wang et al., Aging (Albany NY), 2019; [115]
	exerts anti-inflammatory effects	Sun et al., Cell Mol Neurobiol, 2023; [116]
Bilberry	activates autophagy	Li et al., Nutrition, 2022; [117]
	counteracts oxidative stress (ROS)	Osada et al., PLoS One, 2017; [118]
	exerts anti-inflammatory effects	Wang et al., Molecules, 2015; [119]
Curcumin	activates autophagy	Jin et al., Exp Ther Med, 2022; [120]
	counteracts oxidative stress (ROS)	Lin et al., Phytomedicine, 2023; [9]

**Table 3:** Preliminary data on the improvement of visual acuity and drusen in AMD patients following specific wavelengths and phytochemicals.

Patient	BCVA		Metamorphopsia		Contrast Sensitivity		Central Thickness (μm)		Drusenoid Area (mm^2^)	
	Pre	Post	Pre	Post	Pre	Post	Pre	Post	Pre	Post
1	20/63	20/50	0.4°	0.4°	2.0	2.0	185	213	7.0	0.7
2	20/80	20/50	0.3°	0.4°	1.8	1.8	208	228	13.1	5.72
3	20/50	20/40	0.4°	0.4°	1.8	1.8	212	210	7.0	3.46

## Data Availability

The data that support the findings of this study are available from the corresponding author upon reasonable request.

## Supplementary Materials

The following supporting information can be downloaded at https://www.mdpi.com/article/10.3390/antiox12061183/s1. Figure S1: Optical Coherence Tomography (OCT) from AMD patients.
