# Supplementary material for: Autophagy Activation Promoted by Pulses of Light and Phytochemicals Counteracting Oxidative Stress during Age-Related Macular Degeneration
Source: Antioxidants (Basel). 2023 May 30;12(6):1183. doi: 10.3390/antiox12061183 (PMC10295222; doi:10.3390/antiox12061183)
Supplement: Supplementary file 1 [file antioxidants-12-01183-s001.zip › antioxidants-2311001-supplementary.pdf]

Supplementary Figure Legend

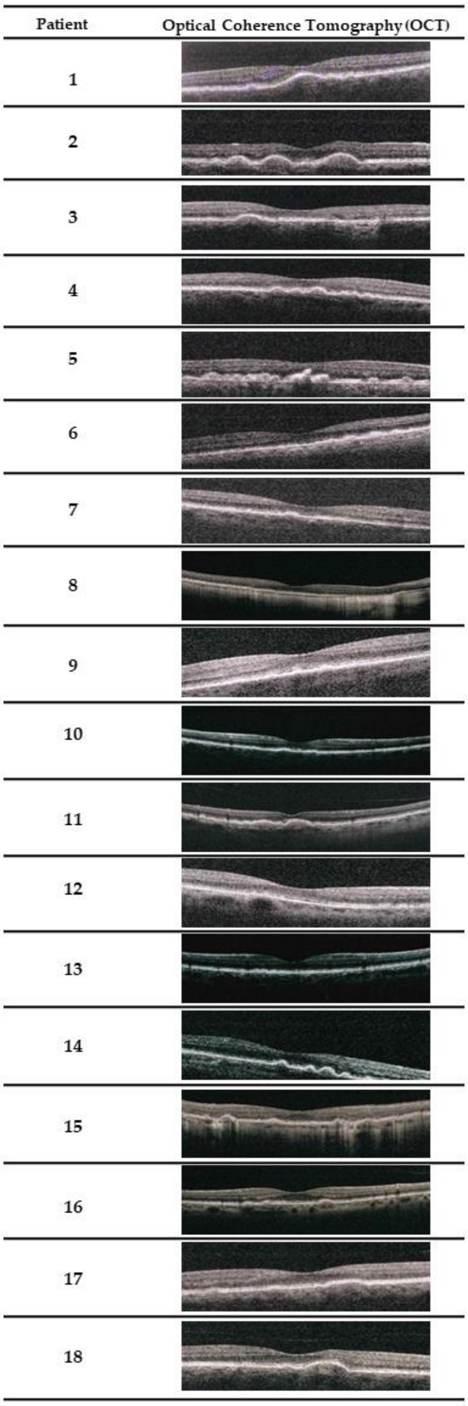

Supplementary Figure S1. Optical Coherence Tomography (OCT) from AMD patients. OCT were obtained from the very same patients (identified by the same progressive number) reported in Table 1, concerning the measurement of visual acuity and the drusenoid area. Theserepresentative OCT confirm the potential dissociation between the loss of visula acuity and the amount of dreusenoid area, which was measured in Table 1. All patients signed an informed consent.
